# Evaluation of GPU‐accelerated Monte Carlo beam and agility MLC modeling in the Monaco treatment planning system

**DOI:** 10.1002/acm2.70839

**Published:** 2026-11-14

**Authors:** Fasco van Ommen, Sima Sajadi Shahrbabaki, Thomas A. Foppen, Jordi Saez, Victor Hernandez, Bram van Asselen, Sara L. Hackett

**Affiliations:** ^1^ Department of Radiation Oncology UMC Utrecht Utrecht the Netherlands; ^2^ Department of Medical Physics & Informatics HollandPTC Delft the Netherlands; ^3^ Department of Radiation Oncology Hospital Clínic de Barcelona Barcelona Spain; ^4^ Department of Medical Physics Hospital Sant Joan de Reus, IISPV Reus Tarragona Spain; ^5^ Cancer Research @UCC University College Cork Cork Ireland

**Keywords:** beam modelling, MLC modelling, Monaco, GPUMCD, VSM2.0

## Abstract

**Background:**

Treatment planning systems (TPSs) are essential in modern radiotherapy. Accurate beam modeling, especially of multileaf collimators (MLCs), is crucial, as their complex designs often introduce systematic uncertainties in planned and delivered doses. Thorough evaluation of the beam model and MLCs must be performed before clinical introduction.

**Purpose:**

This study evaluates a new GPU‐accelerated Monte Carlo (MC) beam and MLC model (VSM2.0) from Elekta, with a focus on aspects of the MLCs that were previously shown to be particularly challenging to model.

**Methods:**

Measurements were conducted on two Elekta Versa HD linacs with Agility MLCs. All calculations were performed in Monaco v6.2.3 using VSM1.6 and VSM2.0. Open field dose distributions were measured and calculated in a water phantom and compared using a gamma analysis (1%/1 mm gamma criterion). MLC characteristics including the leaf tip penumbra, measured with film, the dosimetric leaf gap (DLG) and the tongue‐and‐groove effect, as measured via sweeping gap fields, were compared with calculations from both beam models. These measurements also highlighted some challenges associated with MLC model verification. Finally, patient treatment plans with a wide range of target volumes and modulation were measured and compared against calculations (gamma analysis: 3%/2 mm gamma criterion).

**Results:**

The VSM2.0 model showed excellent agreement with measurements for open field dose distributions, with gamma pass rates for almost all fields above 90%. The clinical treatment plans showed gamma pass rates above 94%. The VSM1.6 model showed similar accuracy. However, the VSM2.0 modeled leaf transmission more accurately than VSM1.6 and improved representation of the tongue‐and‐groove effect across different gap widths and offsets. By contrast, the maximum deviations from the measured DLGs were 0.15 mm and 0.43 mm for VSM1.6 and 2.0 respectively, largely due to user‐definable tuning of parameters for VSM1.6, which allowed the VSM1.6 model to closely match linac performance.

**Conclusions:**

The new GPU‐based single phase‐space beam model shows excellent agreement with measurements and improves simulation of complex aspects of the leaf geometry. Reducing customization simplifies the commissioning process of planning systems, thereby potentially reducing associated risks, but the possibility to tune the leaf offset per beam model may further improve the model accuracy.

## 1 INTRODUCTION

Treatment planning systems (TPSs) are integral to modern external photon beam radiotherapy for creating complex treatment plans. Accurate dose calculations are essential for treatment planning and beam delivery.^1^ Modern TPSs use model‐based algorithms, including collapsed cone convolutions, Linear Boltzmann Transport Equations and Monte Carlo (MC) calculations.^2^ MC methods can provide highly accurate and sufficiently fast dose calculations and are implemented in several commercially TPSs.^1^ However, their computational demands require simplifications of the complex machine and patient models, which can result in deviations between calculated and delivered doses.

Accurate modeling of the beam and beam‐limiting devices, particularly the multileaf collimator (MLC), is necessary as these factors contribute substantially to uncertainties in dose distributions of highly modulated treatment plans.^3^ However, MLC modelling is challenging because of the complexity of MLC designs.^4^ Features such as rounded edges, eccentric curvature of the leaf tip, but also defocused leaf sides complicate the modeling of the Agility MLC.^5^ Previous studies have demonstrated that more sophisticated MLC models can improve dose calculations accuracy in Eclipse,^6^ while inaccuracies in MLC modelling can systematically affect planned versus measured dose. Even when MC is used for dose calculation, MLC models are often simplified and can compromise the overall accuracy.^7^

Agility MLCs are used on most Elekta linear accelerators. The Agility consists of 160 interdigitating leaves with a projected width of 5 mm at isocenter. The leaves are relatively thick, resulting in a transmission below 0.5% and have a tilted design with defocused leaf sides that produces an effective tongue and groove effect. The leaf tips also have an eccentric curvature.^8^ These features require simplifications in TPS implementation. A previous study showed that both RayStation and Monaco could not accurately model the rounded leaf tips or the tongue and groove effect.^5^

Monaco MC TPS (Elekta, Stockholm, Sweden) currently utilizes a three‐component virtual beam source (VSM1.6). Beam‐shaping devices, including the primary collimator, jaws, and MLC are modelled using transmission probability filters (TPFs), which describe the probability of photon transmission through different MLC regions.^1^, ^9^ TPFs include both geometric and transmission parameters and can be configured separately for each beam energy. Relevant geometrical parameters include MLC position offset and the leaf groove width, while transmission parameters include MLC and jaw transmission, interleaf leakage and leaf tip leakage. Despite these configurable parameters, the implementation simplifies the interaction of the MLCs with the radiation beam, as characteristics such as the MLC penumbra depend on the off‐axis position of the MLC. TPFs are initially provided by the vendor but can be optimized by the user. However, there is no standardized process for verifying or modifying the MLC model resulting in substantial variations between institutes for similar equipment and beam energies.^10^

Recently, Elekta has introduced a new GPU‐accelerated MC method for external photon beam calculations based on GPUMCD.^11^ The new beam model (VSM2.0) uses a single phase‐space source and a detailed three‐dimensional MLC model instead of TPFs.^12^ Unlike VSM1.6, VSM2.0 has no user‐configurable parameters and therefore cannot be tuned to measured data. A previous study demonstrated, improved accuracy and consistency for highly complex treatments compared to the previous model.^13^ However, given the previously reported challenges in MLC modelling, a detailed evaluation of the VSM2.0 beam and particularly MLC model is warranted. This study therefore evaluates the new GPU‐based beam and MLC models, focusing on MLC characteristics that are particularly challenging to model.

## 2 METHODS

All calculations were performed in Monaco v6.2.3 (Elekta, Stockholm, Sweden) using our clinical beam model (VSM1.6) and the new beam model (VSM2.0). VSM1.6 was originally created by Elekta, and the TPFs for MLC and jaw were later refined from the default parameters using the tests described by Roche et al.^9^ The parameters are provided in Table S1. VSM2.0 model was created by Elekta from measured profiles and a geometrically accurate model of the linac head and cannot be modified by the user.

MC calculations were performed with a voxel size of 1.0 mm^3^ and a statistical uncertainty of 0.5% per calculation, corresponding to the maximum voxel uncertainty in the high‐dose region, unless specified otherwise. Measurements were performed on Versa HD linear accelerators (Elekta, Stockholm, Sweden) equipped with Agility MLCs and flattened 6 MV and 10 MV and a flattening‐filter‐free 6 MV (6 MV‐FFF)). Agility MLC linacs only have primary jaws, so the field edges are delimited by MLCs in the cross‐beam direction. Unless specified, measurements and calculations were performed for all three energies on two beam‐matched linear accelerators. Water tank measurements were performed using the Blue Phantom^2^ (IBA Dosimetry, Schwarzenbruck, Germany), filled to a depth of 35 cm of water. Other measurements were performed in an RW3 phantom (PTW, Freiburg, Germany). In Monaco, a 50 × 50 × 35 cm^3^ water tank phantom (relative election density (RED): 1.000) and a 30 × 30 × 20 cm^3^ RW3 phantom (RED 1.024) were created.

### 2.1 Open field dose distributions

Output factors (OFs), percentage dose depth curves (PDDs) and dose profiles were measured using a MicroDiamond (PTW, Freiburg, Germany) for small fields (1.0–5.0 cm) and an ionization chamber (CC13, IBA Dosimetry, Schwarzenbruck, Germany) for larger fields (≥ 10 cm). Small field measurements were corrected according to TRS‐483.^14^ Field sizes ranged from 1.0 × 1.0 cm^2^ to 30 × 30 cm^2^ at a source‐to‐surface distance (SSD) of 90 cm. For OF measurements, the chamber was positioned at 10 cm depth on the central axis (200 MU using 10 × 10 cm^2^ field to normalize). For dose profiles were measured at several depths of maximum dose (1.6, 1.8 and 2.0 cm for 6 MV, 6 MV‐FFF and 10 MV, respectively), and 5.0, 10, 20 and 30 cm. The same OFs and dose profiles were calculated for both beam models. Measured and calculated dose distributions were compared using a local gamma analysis with a criterion of 1%/1 mm, using measurements as the reference. No dose threshold was applied to deliberately include the penumbra and out‐of‐field dose. Gamma pass rate and median gamma were determined. Agreement within 1% was required for OFs for fields ≥2.0 × 2.0 cm^2^, and a gamma pass rate > 90% was required.^1^

### 2.2 MLC modeling

#### 2.2.1 Leaf transmission

MLC leaf transmission (considering both intra‐ and inter‐leaf transmission) was measured with collimator and gantry angles 0°. The jaws were completely open and the MLC leaf bank was closed to 15 cm off‐axis. An electrometer (UNIDOS webline, PTW, Freiburg, Germany) and an ionization chamber (30013 Farmer Chamber, PTW, Freiburg, Germany) measured the dose on the central axis at 10 cm depth in a water tank. The chamber was oriented perpendicular to the leaf motion. A total of 1000 MU was delivered at an SSD of 90 cm. Transmission was calculated as the ratio between the dose of the closed MLC field to that of the dose of a 10 × 10 cm^2^ reference field. Each energy was measured twice. For the beam models, transmission was calculated similarly by comparing the dose in a 2.0 mm diameter spherical volume of interest (VOI) for a 1000 MU 10 × 10 cm^2^ field with the same set‐up to the closed‐MLC field.

#### 2.2.2 Leaf tip position and penumbra

Leaf tip position and penumbra measurements require high spatial resolution, necessitating the use of GafChromic EBT3 film^5^ (Ashland Inc., Covington, USA). A 20‐point calibration curve was generated for each film batch using known doses under reference conditions. Films were scanned approximately 24 h after irradiation and converted to dose using the calibration curve and in‐house software. A 1 × 24 cm^2^ field was irradiated on the central axis on film at 5 cm depth in RW3 (SSD of 95 cm,200 MU) to evaluate the leaf tip model. The same configuration was calculated for both beam models. Profiles were averaged over 1 cm perpendicular to the leaf motion to reduce noise. Effective leaf tip position was assessed from the 50% dose point.^7^ Penumbra (20 ‐ 80%) was also visually inspected, particularly the inflection points.

#### 2.2.3 Leaf tip offset

Three abutting fields (5 × 20 cm^2^) were irradiated on GafChromic EBT3 film (Ashland Inc., Covington, USA) at 5 cm depth in RW3 (SSD of 95 cm, 200 MU per field) to assess the offset between the nominal and actual leaf tip position in VSM1.6 and VSM2.0. An MLC calibration check was performed before irradiation showed an average relative positional error of the MLCs of 0.18 mm across the leaf bank. Film measurements were registered to Monaco dose profiles calculated with the same field configuration. Profiles were averaged over 1 cm perpendicular to leaf motion, and visual inspection of the profiles was performed.

#### 2.2.4 Dosimetric leaf gap

Dosimetric leaf gap (DLG) was determined using sweeping gap (SG) fields.^15^ Five SG fields with gaps of width *g* of 5.0, 10, 20, 30 and 40 mm and a travel length of 10 cm were irradiated using 200 MU each. Dose was measured at 5 cm depth at an SSD of 90 cm using a Farmer type ionization chamber (30013 Farmer Chamber, PTW, Freiburg, Germany) on the central axis in RW3. A 10 × 10 cm^2^ field was used for normalization. For both beam models, dose was calculated using a 2.0 mm diameter spherical VOI at 5 cm depth with the same configurations. DLGs were calculated as described previously^15^: normalized measured charge, or calculated dose was corrected for leaf transmission and leaf travel, followed by linear fitting of the five SG measurements. DLG was defined as the additive inverse of the intersection of the fit with the horizontal axis of the gap.

#### 2.2.5 Tongue and groove effect

Although Agility MLCs lack a physical tongue‐and‐groove, their tilted leaves nonetheless produce an effective tongue‐and‐groove effect. Dynamic asynchronous sweeping gap (aSG) fields were configured according to Hernandez et al.,^16^ SG fields were created with gap width *g*, but alternate adjacent leaves were offset by a distance *s* to expose the sides of the leaves. Larger *s*‐values increase transmission through the tongue‐and‐groove regions. For each gap *g*, the *s‐*values ranged between 0 and up to 2 *g*. For both VSM models, aSG fields were calculated at 6 MV in RW3 at an SSD of 90 cm. A 4.0 × 2.0 × 1.0 cm^3^ VOI was positioned on the central axis at 10 cm depth, with the long axis perpendicular to leaf motion, to determine mean dose per field. Leaves travelled 12 cm and each field delivered 200 MU. To obtain sufficient dose precision in Monaco, calculations used 50 fractions, a 2.5 mm grid and 0.2% statistical uncertainty per control point. Calculations for 5.0, 10 and 20 mm gaps were repeated using a 1.0 mm grid; differences were < 0.1%, so larger gaps were not repeated.

Fields were exported from Monaco and imported in Mosaiq v3.2.1.3. Measurements used an ionization chamber (30013 Farmer Chamber, PTW, Freiburg, Germany) and UNIDOS webline electrometer (PTW, Freiburg, Germany) in RW3 with an SSD of 90 cm. Each field was measured twice with the chamber perpendicular to the leaf travel direction; once with the chamber positioned +2.125 cm and −2.125 cm, that is with a 4.25 cm shift in the in‐plane direction to better estimate the average dose across multiple leaves. Measured and calculated doses were normalized relative to the reading at the isocenter of a 10 × 10 cm^2^ field for 200 MU. The tongue‐and‐groove effect, *Δφ*_TG_(*s*), was calculated using Equation 10 ($\Delta \varphi_{TG}\;\left(s\right)=\frac{D_{SG}-\;D_{aSG}}{2k}$, where $D_{SG}$ is the average dose for a synchronous SG of a certain gap size (s), $D_{aSG}$ is the average dose for an aSG of the same gap size and distance between adjacent leaves equal to *s* and k is a constant that relates fluence values to dose units) and the results fitted with Equation 11 ($\Delta \varphi_{TG}\;\left(s\right)=a_{1}\;*s-a_{2}\left(1-e^{-a_{1}*s/a_{2}}\right)$) given in Hernandez et al.^5^ Fits were performed in MATLAB R2019A, and the resulting fit of Equation 11 to each of the *Δφ*_TG_(*s*) curves yielded parameters *a*_1_ and *a*_2_ that were then used to plot the first derivative of the fit.

### 2.3 Patient plans

For each energy, ten recent clinical VMAT plans were reevaluated using both VSM models. Plans represented a range of target sizes, treatment sites (including breast, brain, prostate, pancreas, lung and esophagus), complexity and modulation levels. All plans were measured on a Delta4 (ScandiDos, Uppsala, Sweden) and compared with TPS calculations. Each plan was recalculated using both VSM1.6 and VSM2.0 and compared with measurements from a single linac. Dose deviation, distance‐to‐agreement (DTA) and global gamma pass rate were evaluated. A gamma pass rate ≥95% was required using a 3%/2 mm criterion and a low‐dose cutoff of 10% of the normalization dose.^17^

## 3 RESULTS

### 3.1 Open field dose distributions

OFs calculated with VSM1.6 and VSM2.0 agreed well with measurements. Differences were within 1% for all field sizes (median difference: 0.3%, range: 0.0%–0.9%), except for the 1 × 1 cm^2^ field where differences reached 5.5% for VSM1.6 and 4.5% for VSM2.0 Gamma passing rates for VSM1.6 and VSM2.0 were consistent (Tables S2–S4), with a slight decrease for larger fields at all energies. This was primarily due to a small deviation at 30 cm depth, where scattering conditions in the water phantom were less accurately modeled. The remainder of the PDDs showed very good agreement with the measurements. Median gamma values were low for both VSM1.6 (average: 0.37, 0.37 and 0.42 for 6 MV, 10 MV and 6 MV‐FFF), and VSM2.0 (average: 0.35, 0.36 and 0.28).

### 3.2 MLC modeling

#### 3.2.1 Leaf transmission

VSM2.0 showed closer agreement with measured MLC transmission than VSM1.6 (Table 1). The maximum absolute difference between measurements on both linacs was <0.1% for all three energies. The maximum absolute difference between VSM 2.0 and measurements was 0.1%. VSM1.6 slightly overestimated transmission 6 MV and 10 MV (<0.2%) and more substantially for 6 MV‐FFF around (∼0.45%).

**TABLE 1:** Average measured MLC transmission (%) and calculated transmission using VSM1.6 and VSM2.0. Both measured values are shown in brackets.

	6 MV	10 MV	6 MV‐FFF
Measurement (%)	0.49 [0.45–0.52]	0.52 [0.47–0.57]	0.33 [0.30–0.37]
VSM1.6 (%)	0.66	0.64	0.79
VSM2.0 (%)	0.58	0.59	0.41

#### 3.2.2 Leaf tip position and penumbra

Both VSM models showed similar discrepancies in the measured out‐of‐field doses, likely due to limitations of film dosimetry in low dose regions and the underestimation of out‐of‐field doses observed for Monaco.^18^ Agreement in the inflection point was high, with differences at the 50% dose below 0.3 mm for both models. Figure 1 shows an example for 6 MV. The maximum voxel uncertainty was 0.5%, by averaging over 10 rows of voxels the profile uncertainty reduced to 0.2%. The estimated maximum uncertainty of the absolute film dose was 2%.^19^

**FIGURE 1:**
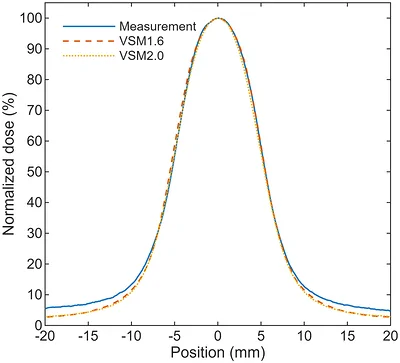
Example crossline profile of the 1 × 24 cm^2^ field of 6 MV, representative of 10 MV and 6 MV‐FFF. Profiles are normalized to maximum dose and averaged over 1 cm to reduce noise. Measurements and both beam models show high agreement in the inflection points, with a difference in the out‐of‐field dose.

#### 3.2.3 Leaf tip offset

For the abutting fields, VSM1.6 showed higher absolute doses than the VSM2.0, but also better agreement in profile shape with measurements. At the field junctions at −25 and + 25 mm, measurements and VSM1.6 showed a dose peak, whereas VSM2.0 showed a minor dose reduction (Figure 2). Similar results were observed for the other energies and linacs. Calculation and measurement uncertainties were as described above.

**FIGURE 2:**
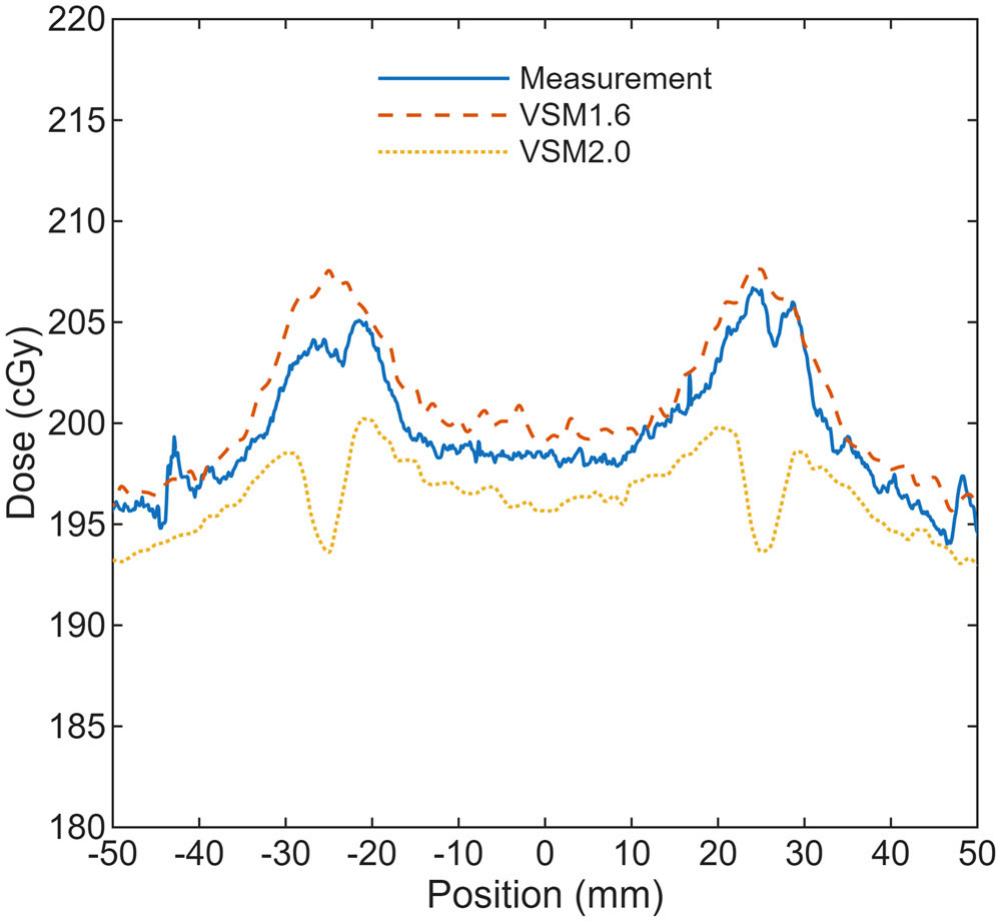
Example crossline profile of abutting fields (3 fields 5 × 20 cm^2^). Measurements and VSM1.6 show a slight increase at the junctions, whereas VSM2.0 shows a minor dip. Profiles were averaged over 1 cm.

#### 3.2.4 Dosimetric leaf gap

VSM1.6 DLGs agreed closely with measurements for all energies, with differences below 0.15 mm. VSM2.0 produced substantially lower DLGs, with differences of 0.29–0.43 mm (Table 2).

**TABLE 2:** Average measured DLG and calculated DLG in mm using VSM1.6 and VSM2.0. The difference between measurement (Diff. Meas) and VSM1.6 and VSM2.0 is also shown.

	6 MV	10 MV	6 MV‐FFF
Measurement (mm)	0.40	0.50	0.49
VSM1.6 (mm)	0.37	0.47	0.34
VSM2.0 (mm)	−0.028	0.21	0.13
Diff. Meas.—VSM1.6 (mm)	0.03	0.03	0.15
Diff. Meas.—VSM2.0 (mm)	0.43	0.29	0.36

#### 3.2.5 Tongue and groove effect

VSM1.6 overestimated dose relative to measurements, whereas VSM2.0 showed excellent agreement across all *s*‐values. Figure 3a, shows the 40 mm gap, representative of the other gap widths. For VSM1.6, *Δφ*_TG_(*s*) agreed well for small *s*‐values, but was underestimated when *s* exceeded the gap width *g*. VSM2.0 showed excellent agreement across all values of *s* (Figure 3b). Furthermore, the tongue‐and‐groove effect increased with exposed leaf edge length and stabilized at around *s *= 20 mm, corresponding to approximately half the sweeping gap width (Figure 3c). Equation 11 from Hernandez et al.,*5* described VSM2.0 results well, but did not adequately represent VSM1.6.

**FIGURE 3:**
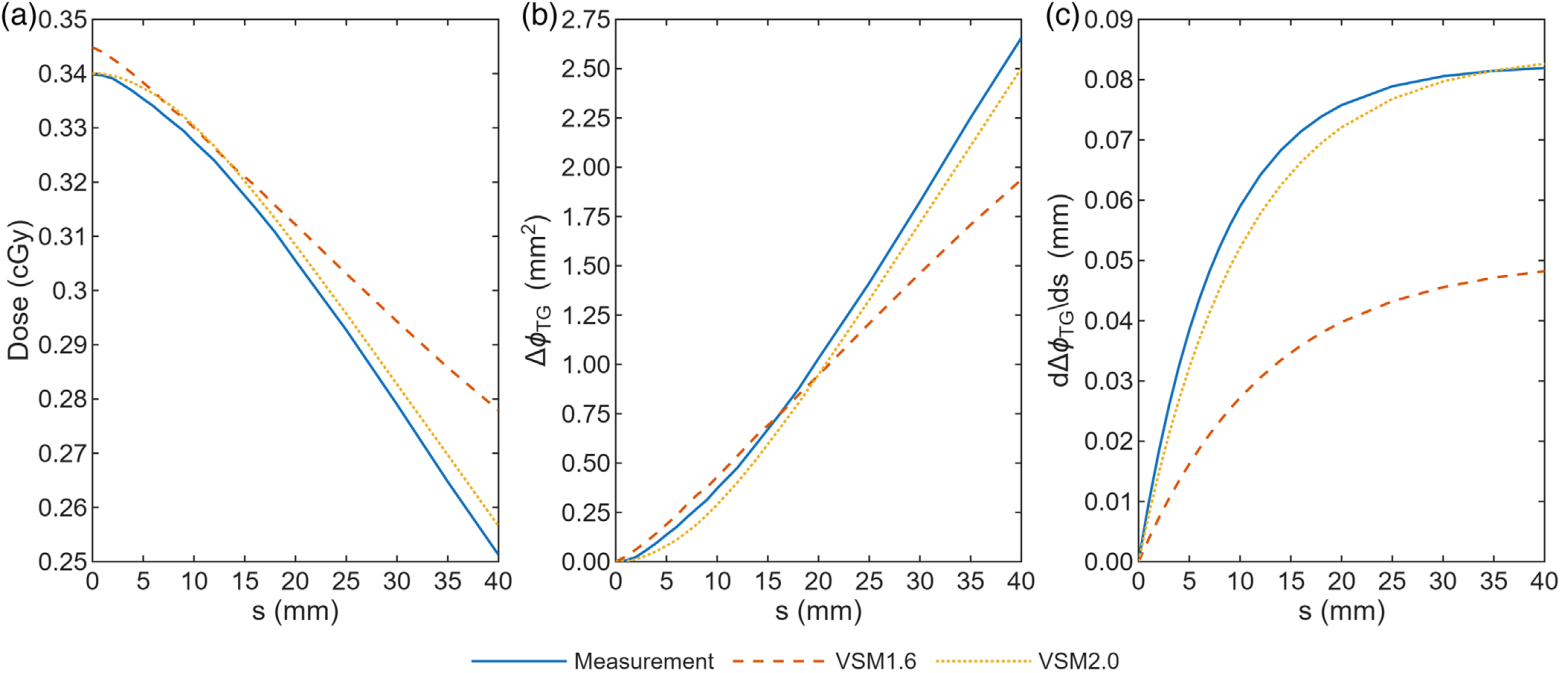
Results for the 40 mm gap aSG experiment. (a) Measured and calculated normalized doses (D(s)). (b) change in fluence due to the tongue and groove effect, and (c) sensitivity of fluence to geometric changes.

### 3.3 Patient plans

Both models showed good agreement with measurements for all 10 patient treatment plans per energy (Table 3). VSM2.0 showed slightly higher dose deviations DTA and gamma passing rate were similar. In two cases, the 95% passing rate was not achieved (94.4%). Both cases had a slightly lower DTA compared to the other clinical plans.

**TABLE 3:** Median dose deviation, DTA passing rate and gamma passing rate for the 10 treatment plans per energy. 6 MV and 10 MV plans were non‐stereotactic; all 6 MV‐FFF plans were stereotactic. Ranges are shown in brackets.

	Model	6 MV	10 MV	6 MV‐FFF
Dose deviation (%)	VSM1.6	0.30 [−0.20–0.90]	0.55 [−0.40–1.10]	0.40 [−1.30–1.50]
	VSM2.0	1.10 [0.80–1.80]	0.55 [−0.30–1.30]	0.45 [0.00–1.40]
DTA (%)	VSM1.6	99.3 [98.5–100]	98.4 [96.0–100]	97.2 [77.7–99.6]
	VSM2.0	97.9 [94.5–99.5]	98.1 [91.4–100]	97.5 [84.9–100]
Gamma (%)	VSM1.6	99.9 [99.2–100]	99.8 [97.7–100]	99.2 [94.4–100]
	VSM2.0	99.7 [94.4–100]	99.9 [98.2–100]	99.3 [97.0–100]

## 4 DISCUSSION

In this study, the new MC‐based beam model (VSM2.0) was examined and its performance was compared to our currently used clinical beam model (VSM1.6) and measurements. The performance of the beam models was evaluated using output factors, open field dose distributions and treatment plans. Careful attention was paid to the model of the Agility leaf tips, as this is a previously identified shortcoming of the MLC model implemented in Monaco for VSM1.6.

VSM1.6 produced DLGs with a close agreement with measured DLGs for all energies, reflecting the result of extensive tuning of the clinical beam model, as improving this parameter has a detectable effect on dose distributions.^15^ The abutting‐field measurements similarly indicated a closer effective MLC offset for VSM1.6, although these measurements, are known to have limited accuracy and agreement for such specific geometry could be misleading, because it could imply discrepancies in average doses for clinical plans. The VSM2.0 DLG results suggest that a small offset should be applied. This would allow fine‐tuning of the calculations to measurements and to the MLC calibration.

VSM2.0 modelled the tongue‐and‐groove effect well, showing very similar behavior to the measured data over the range of sweeping gap widths and *s*‐values. Previous work assessed this effect using a diode array to measure the dose distribution from a FOURL test.^13^ However, these tests measure the dose several centimeters from the leaf tip and therefore do not assess the effects of the rounded leaf tip.^5^ In addition, diode array resolution may underestimate the dose minima. Our results showed that VSM1.6 has good agreement with measurements for low *s*‐values but increasingly underestimates the tongue‐and groove effect for high *s*‐values. This is consistent with previous observations that the effective tongue‐and‐groove width of the Agility MLC increases progressively up to 20 mm from the leaf tip before stabilizing.^5^, ^13^ The fixed leaf‐groove width in VSM1.6 TPF cannot adequately reproduce this behavior, whereas VSM2.0 can accurately simulate the complex impact of this effect.

VSM2.0 modelled average MLC transmission more accurately compared to VSM1.6, especially for 6 MV‐FFF. Both models showed similar high agreement for leaf‐tip penumbra. VSM1.6 appears to model the leaf position slightly more accurate, which is expected as this parameter was previously tuned against measurements. The MLC calibration measurements indicated a small relative leaf position error that should also be considered when interpreting the observed offset.

Both VSM1.6 and 2.0 showed excellent agreement for open field dose distributions and high gamma passing rates for clinical plans. These results show that VSM2.0 can maintain a similar performance compared to VSM1.6. The absence of a measurable improvement in clinical‐plan QA is not unexpected, as VSM1.6 was extensively optimized. Furthermore, the detectability of MLC modelling errors with QA measurements is questionable.^7^

An important advantage of VSM2.0 is that it does not require tuning, which could simplify commissioning efforts and reduce inter‐institutional variability in MLC parameters used by the community.^20^ The disadvantage is the inability to modify the model if discrepancies are identified. The small inter‐linac variation observed suggests that the beams and Agility MLCs are sufficiently well matched to support a single, non‐adjustable model. Nevertheless, the DLG measurements and small systematic dose deviation in clinical plans suggest that a single tunable leaf‐position offset parameter could further improve agreement, which most likely would be a fine tuning within ± 0.2 mm. Such adjustment may be particularly beneficial for small target volumes and highly modulated plans containing small apertures.

The main distinction between VSM1.6 and VSM2.0 is therefore not overall clinical plan performance but the modelling approach. VSM1.6 permits parameter tuning to match measurements, whereas VSM2.0 is designed to realistically represent MLC characteristics through explicit radiation transport within the MLC without user‐adjustable parameters. VSM2.0 provides a balanced representation of MLC characteristics, particularly the DLG and tongue‐and‐groove effect. Although the DLG agreement is slightly less accurate than for VSM1.6, this is compensated by improved modelling of the tongue‐and‐groove effect, both near and further from the leaf tip. The DLG should therefore not be considered independently; the overall MLC model determines the resulting dose distribution.

This study has several limitations. Firstly, not all measurements were performed on each linac on the same day, introducing potential day‐to‐day output variation. This was largely mitigated by relative or corrected measurements, although small effects may remain for absolute measurements. Secondly, the dose‐deposition engines differ between models: VSM1.6 uses XVMC, whereas VSM2.0 GPUMCD. Previous studies have demonstrated high accuracy of both algorithms, particularly in water, and comparable accuracy when beam models are appropriately fitted to measurements. The systematic differences observed here are therefore more likely attributable to the beam and MLC models than the dose‐deposition algorithms.^11^

## 5 CONCLUSION

The new single phase‐space MC‐based beam model from Elekta for treatment planning with the Elekta Agility MLCs shows high agreement with measurements and with the current three‐component beam modeling approach. The inability of user specific beam modelling optimization is acceptable due to highly accurate representation of the Agility MLCs with the new VSM2.0, which will reduce variability between institutes, streamlining the commissioning process and, ultimately, improving safety. However, a single tunable parameter for the leaf positioning offset may further improve the accuracy of the MLC model.

## AUTHOR CONTRIBUTIONS

**Fasco van Ommen**: Data collection; analysis; manuscript draft; manuscript correction and submission. **Sima Sajadi Shahrbabaki**: Data collection; manuscript correction and manuscript approval. **Thomas A. Foppen**: Data collection; manuscript correction and approval. **Jordi Saez**: Analysis; manuscript correction and manuscript approval. **Victor Hernandez**: Analysis; manuscript correction and manuscript approval. **Bram van Asselen**: Manuscript correction and manuscript approval. **Sara L. Hackett**: Data collection; analysis; manuscript draft; manuscript approval and supervision.

## CONFLICT OF INTEREST STATEMENT

The authors declare no conflicts of interest.

## ETHICAL APPROVAL

This study did not involve human participants, human tissue, or identifiable patient data. Therefore, Institutional Review Board approval and informed consent were not required.

## Supporting information




Supporting Information

Supporting Information

Supporting Information

Supporting Information

## Data Availability

The data used and generated in this work may be available under data protection considerations upon request to the leading institution on an individual basis.
