# Supplementary material for: Evaluation of GPU‐accelerated Monte Carlo beam and agility MLC modeling in the Monaco treatment planning system
Source: J Appl Clin Med Phys. 2026 Nov 14;27(11):e70839. doi: 10.1002/acm2.70839 (PMC13769378; doi:10.1002/acm2.70839)
Supplement: Supplementary file 1 [file ACM2-27-e70839-s001.docx]

**Table S1**: Default and current MLC and jaw parameters for the VSM1.6 beam models for the three different energies. Changed parameters are highlighted bolt.

| Parameter | Default | 6MV | 10MV | 6MV-FFF |
| --- | --- | --- | --- | --- |
| Static leaf gap (mm) | 0.10 | 0.10 | 0.10 | 0.10 |
| Leaf transmission (%) | 0.0030 | **0.0032** | **0.0027** | **0.0050** |
| Interleaf leakage | 3.0 | **7.0** | **7.0** | 3.0 |
| TJaw transmission (%) | 0.0050 | **0.0037** | **0.0034** | 0.0050 |
| TJaw tip leakage | 1.03 | 1.03 | 1.03 | 1.03 |
| Leaf offset (mm) | 0.00 | **-0.06** | **-0.05** | 0.00 |
| Leaf tip leakage | 1.10 | **1.18** | **1.18** | **1.18** |
| MLC leakage | 0.00 | 0.00 | 0.00 | 0.00 |
| Leaf groove width (mm) | 0.40 | 0.40 | 0.40 | 0.40 |
