# Supplementary material for: Evaluation of GPU‐accelerated Monte Carlo beam and agility MLC modeling in the Monaco treatment planning system
Source: J Appl Clin Med Phys. 2026 Nov 14;27(11):e70839. doi: 10.1002/acm2.70839 (PMC13769378; doi:10.1002/acm2.70839)
Supplement: Supplementary file 2 [file ACM2-27-e70839-s002.docx]

**Table S3**: Gamma passing rates and median gamma values for 10MV for PDDs and profiles of field sizes ranging from 1 – 30 cm.

|  | Gamma (%) |  | Median |  |
| --- | --- | --- | --- | --- |
| Field size | VSM1.6 | VSM2.0 | VSM1.6 | VSM2.0 |
| 1.0x1.0 | 73.5 | 65.3 | 0.59 | 0.79 |
| 2.0x2.0 | 96.4 | 96.0 | 0.26 | 0.29 |
| 3.0x3.0 | 92.5 | 93.1 | 0.29 | 0.29 |
| 5.0x5.0 | 87.2 | 88.0 | 0.44 | 0.40 |
| 10.0x10.0 | 95.6 | 97.9 | 0.28 | 0.23 |
| 15.0x15.0 | 93.5 | 97.9 | 0.30 | 0.23 |
| 20.0x20.0 | 93.2 | 95.8 | 0.35 | 0.27 |
| 30.0x30.0 | 87.5 | 86.4 | 0.46 | 0.42 |
| Average | 89.9 | 90.1 | 0.37 | 0.36 |
