# Supplementary material for: Evaluation of GPU‐accelerated Monte Carlo beam and agility MLC modeling in the Monaco treatment planning system
Source: J Appl Clin Med Phys. 2026 Nov 14;27(11):e70839. doi: 10.1002/acm2.70839 (PMC13769378; doi:10.1002/acm2.70839)
Supplement: Supplementary file 3 [file ACM2-27-e70839-s003.docx]

**Table S4**: Gamma passing rates and median gamma values for 6MV- FFF for PDDs and profiles of field sizes ranging from 1 – 30 cm.

|  | Gamma (%) |  | Median |  |
| --- | --- | --- | --- | --- |
| Field size | VSM1.6 | VSM2.0 | VSM1.6 | VSM2.0 |
| 1.0x1.0 | 91.6 | 90.2 | 0.40 | 0.32 |
| 2.0x2.0 | 94.5 | 96.9 | 0.36 | 0.17 |
| 3.0x3.0 | 92.6 | 93.1 | 0.37 | 0.25 |
| 5.0x5.0 | 95.0 | 95.7 | 0.31 | 0.25 |
| 10.0x10.0 | 97.6 | 97.3 | 0.18 | 0.21 |
| 15.0x15.0 | 88.8 | 96.1 | 0.31 | 0.24 |
| 20.0x20.0 | 81.1 | 93.0 | 0.41 | 0.28 |
| 30.0x30.0 | 55.0 | 84.7 | 1.07 | 0.49 |
| Average | 87.0 | 93.4 | 0.42 | 0.28 |
