# Supplementary material for: Evaluation of GPU‐accelerated Monte Carlo beam and agility MLC modeling in the Monaco treatment planning system
Source: J Appl Clin Med Phys. 2026 Nov 14;27(11):e70839. doi: 10.1002/acm2.70839 (PMC13769378; doi:10.1002/acm2.70839)
Supplement: Supplementary file 4 [file ACM2-27-e70839-s004.docx]

**Table S2**: Gamma passing rates and median gamma values for 6MV for PDDs and profiles of field sizes ranging from 1 – 30 cm.

|  | Gamma (%) |  | Median |  |
| --- | --- | --- | --- | --- |
| Field size | VSM1.6 | VSM2.0 | VSM1.6 | VSM2.0 |
| 1.0x1.0 | 73.8 | 71.0 | 0.46 | 0.66 |
| 2.0x2.0 | 83.5 | 83.6 | 0.34 | 0.37 |
| 3.0x3.0 | 96.3 | 97.2 | 0.22 | 0.18 |
| 5.0x5.0 | 93.1 | 97.6 | 0.28 | 0.22 |
| 10.0x10.0 | 94.0 | 98.3 | 0.24 | 0.16 |
| 15.0x15.0 | 96.2 | 97.3 | 0.30 | 0.22 |
| 20.0x20.0 | 89.8 | 86.7 | 0.39 | 0.37 |
| 30.0x30.0 | 65.0 | 71.6 | 0.73 | 0.60 |
| Average | 86.5 | 87.9 | 0.37 | 0.35 |
